# The layering construction of the three-dimensional (3D) geological model for Wudalianchi volcanic area, Northeast China

**DOI:** 10.1371/journal.pone.0315326

**Published:** 2025-02-11

**Authors:** Xue Jiang, Ranran Li

**Affiliations:** 1 School of Water Conservancy and Civil Engineering, Northeast Agricultural University, Harbin, China; 2 Key Laboratory of High Effective Utilization of Agricultural Water Resources, Ministry of Agriculture and Rural Affairs, Northeast Agricultural University, Harbin, China; China University of Mining and Technology, CHINA

## Abstract

Establishment of geological model in volcanic area is challenging owing to lack of borehole data and the effect of volcanic activity on rock distribution. Taking full advantage of the existing complete volcanic landforms and recognized for seven eruptive cycles in Wudalianchi volcanic area, here we apply a layered approach to build geological models for meeting rapid development of agriculture and understanding the evolution of regional geological structure. Based on the volcanic eruption cycle, the stratas in Wudalianchi volcanic area are divided into four layers. UGrid (unstructured grid in GMS) is used combining with DEM data to hierarchically build 3D geological structure model of volcanic area, which realize the visualization of regional stratigraphic distribution, and the reliability of the model is verified by the formation mechanisms of different types springs. The stratified modeling provides a scientific and effective mean for the reconstruction of geological structure in volcanic areas where the data are short and the stratigraphic distribution is complex. The 3D geological structure model established can lay a foundation for the prediction, evaluation and sustainable use of regional groundwater, geothermy, mineral water and mineral mud resources.

## 1. Introduction

3D geological structure model is a visualization study to reflect geological phenomena based on the simulation of regional strata distribution. Reconstruction of the underground data plays a vital role in urban planning [1], engineering decision-making [2], geological analysis [3,4], automatic mapping [5] and groundwater resource evaluation and simulation [6,7]. Therefore, 3D geological modeling has been widely used in geology, mining, geotechnical and hydrogeological engineering and so on. However, due to the heterogeneity of strata and the limitation of exploration technology, data obtained from geological exploration are usually sparse, uneven and incomplete. How to build a 3D visualization model approximating the real strata distribution with limited data is a hit as well as difficulty in current research [8]. Especially for polyphasic volcanoes that have erupted many times in history, whose strata distribution is much more complex than that in sedimentary areas. Moreover, it's difficult and complicated to exploit boreholes to get geological data, which make it far harder to establish 3D geological structure model. The Wudalianchi volcano group is divided into seven eruption cycles, each of which erupts new lava flows to form a lava platform with a certain thickness. Multiple eruption cycles are bound to lead to the accumulation of multiple lava platforms, and it is this formation mechanism that creates the conditions for layered modeling. Layered modeling can make full use of all formation data, and it is convenient to modify the model, so as to realize the authenticity and flexibility of modeling. The layered modeling based on eruption cycle provides a scientific and effective way to reconstruct the geological structure of volcanic areas with multiple eruptions, and also provides a possibility for the 3D geological modeling of data-lacking regions.

Methods for 3D geological modeling can be divided into four categories: geostatistical approach [9–11], geophysical approach ([12–14], software modeling approach. The software mainly include Leapfrog Geo [15], GIS [16,17], GoCAD [18,19], GMS [20]. The last approach is fusion technology of multi-source data, which comprehensively uses geology, drilling, geography and other data sources to build a 3D geological model, so as to make up for the shortage of single data source and make the model more in line with the actual geological situation. This study combines the borehole data, geological point data, DEM and field survey to construct the geological structure model of Wudalianchi volcanic area, aiming to reconstruct real geological conditions and reduce the uncertainty of data by more geological control points [21,22].

Groundwater Modeling System (GMS) is well known as a numerical simulation software for groundwater flow and quality, but in practice, it can also be used to model geological structures. TIN [23,24] is the most widely used form of spatial data in the construction of geological structures in former researches. This paper adopts the latest UGrid [25] rather than TIN to build the model, mainly because UGrid allows for more realistic model of geologic features such as pinchout, which is particularly important in geological modeling of volcanic area. The stratigraphic distribution in Wudalianchi volcanic area is uniform under the effect of multiple cycles of volcanic eruption. Meanwhile, data type can be transformed from Scatter points to TIN and then to UGrid, which provides the possibility for layered modeling. The layered modeling method has the characteristics of flexible application, easy modification and improvement. The validation of 3D geological structure model is much more difficult than that of flow model [26,27]. As it needs more data to build the model, there may be no enough data left to vertify the model, so few researchers verified their 3D geological structure model, only simply explained through comparation, correlation analysis, geological information and so on [28,29]. In this paper, the profiles of different types springs are selected to verify the model through the formation mechanism of springs, which greatly improve the reliability of the model.

On the basis of previous studies, the stratified modeling method is used to reconstruct the geological structure of Wudalianchi volcanic area, which fills in the blank for 3D geological modeling of complex volcanic region.

## 2. Geological background

**Fig 1:**
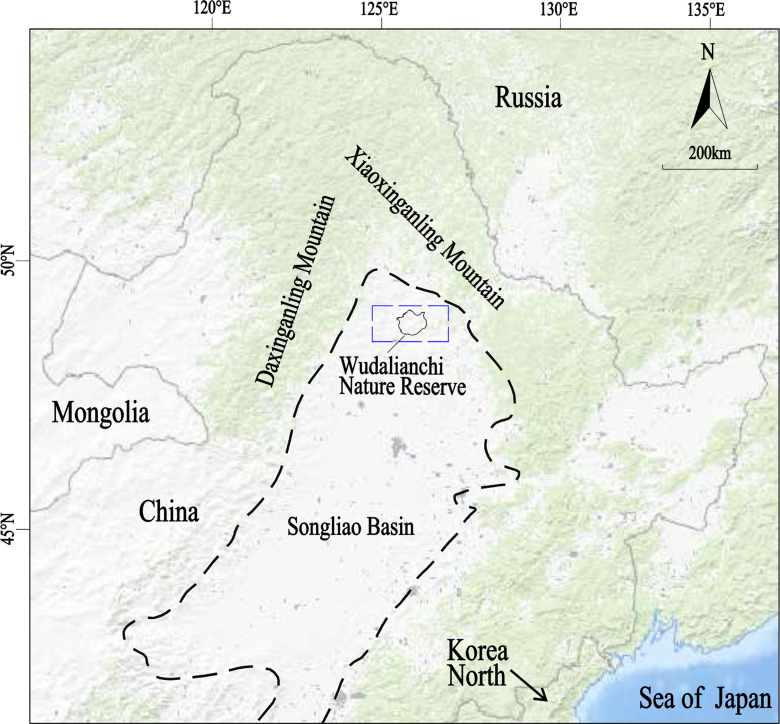
Location map of Wudalianchi Nature Reserve.

**Fig 2:**
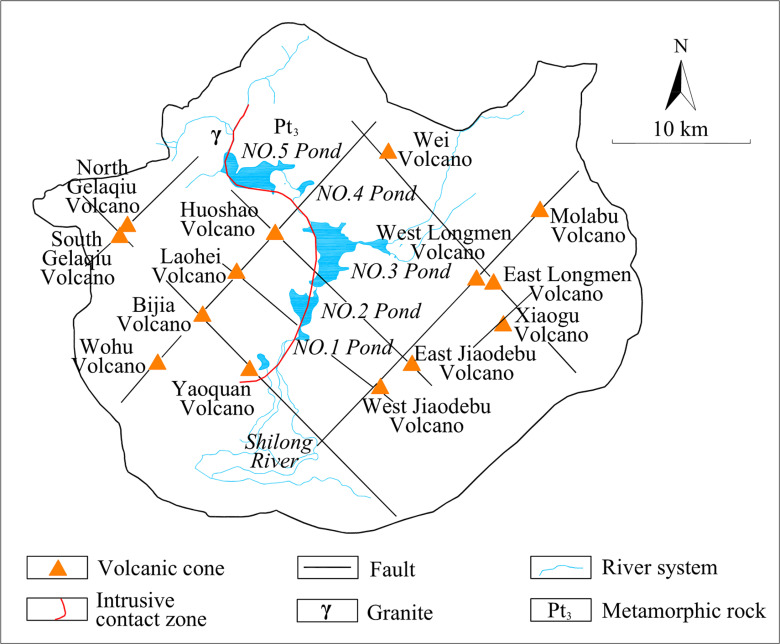
Structure map of Wudalianchi Nature Reserve.

Wudalianchi Nature Reserve (Fig 1) is located in the transition zone from the Xinganling Mountain to the Songnen Plain, with Songliao fault depression basin in the south and Xiaoxinganling fault uplift area in the north. It's higher in the east, north and west and lower in the center and south, with the highest elevation of 600 m, and the lowest of 248 m. Geomorphology belongs to the gentle basalt platform. The volcanic landforms in the area are well developed, including volcanic cones, lava platforms and volcanic barrier lakes (five pools, etc.), as shown in Fig 2. The enrichment of regional surface water and the circulation of groundwater are mainly controlled by unique topography and geomorphology. The unique topography is mainly reflected in two aspects. Firstly, sedimentary metamorphism combined with multi-stage volcanic eruptions result in diverse rock formations. Secondly, volcanic eruptions lead to dramatic changes in the thickness of rock layers and more significant heterogeneity. Therefore, it is necessary to choose a suitable method for reconstructing the regional geological model in order to provide theoretical and technical support for the prediction of regional water resources, mineral mud resources and geothermal resources.

### 2.1. Strata

The stratas of Wudalianchi volcanic area are mainly composed of Lower Cambrian, Upper Cretaceous and Quaternary System (Table 1).

**Table 1.** Stratigraphic summary of Wudalianchi volcanic area.

System	Series	Rock stratigraphic units	Thickness (m)	Area (km^2^)	Rock Types	Depositional environment or sedimentary facies
Quaternary System	Olocene	Laoheishan Formation	41.78	68.15	rhyakkumulate lumps, pu-mice clasts, volcanic bombs and basalt	terrestrial facies, volcanic explosive facies, effusive facies, intrusive facies
		Low Floodplain Deposits Bed	4.80	137.48	gravel and silt	modern fluvial proluvial facies
		High Floodplain Deposits Bed	6.80	161.12	silt, gravel, pebbly Grit, mucky silty clay, silty clay	
	Upper Pleistocene	Guxiangtun Formation	11.50	33.50	gravel, mucky silty clay, peat, paleosols, silty clay	fluvial (second bottom) facies
		Harbin Formation	18.35	368.36	sandy silty clay, silty clay	periglacial alluvial diluvial overlying facies
	Middle Pleistocene	Bijiashan Basalt	53.65	49.55	rhyakkumulate lumps, pumice clasts, volcanic bombs and basalt	terrestrial facies, volcanic explosive facies, effusive facies
		Weishan Basalt	77.77	312	pumice clasts, volcanic bombs, volcanic cinder, rhyakkumulate lumps and basalt	terrestrial facies, volcanic explosive facies, effusive facies
		Upper Huangshan Formation	10.80	concealed	gravel, fine sandstone, silty clay with Iron strips	diluvial overlying facies
		Jiaodebu Basalt	61.10	13.8	pumice clasts, volcanic cinder, thin layers of rhyakkumulate lumps and basalt	terrestrial facies, volcanic explosive facies, effusive facies
	Lower Pleistocene	Lower Huangshan Formation	7.50	concealed	gravel, mucky silty clay	fluvial (second bottom) facies
		Dongshenjing Formation	15.30	concealed	mucky silty clay, silty clay, carbonaceous silt, silty clay	lacustrine facies
		Gelaqiushan Basalt	58.30	30.85	Thin Layers Of Fused Breccis Lumps	terrestrial facies, volcanic explosive facies, effusive facies
		Lynx Formation	9.60	0.04	Gravel, Medium-Fine Sandstone	fluvial facies
Upper Cretaceous System	Upper Cretaceous	Upper Neniiang Formation	114.17	153.90	medium and fine-grained Sandstones, mudstone, silty mudstone, fine sandstone, pebbled gritty sandstone conglomeratic sandstone	lacustrine facies
		Lower Nenjiang Formation	232.78	33.50	conglomeratic sandstone, argillaceous conglomerates, mudstone, silty mudstone contain a large number of conchostracans and ostracodes	hallow lagoon facies
Lower Cambrian System	Lower Cambrian	Beikuanhe Formation	244.82	0.01	silt Slates, carbonaceous slates, biotite Schist, two-mica schist	epicontinental sedimentary metamorphic facies

*Note: According to Bureau of Geology and Mineral Resources in Heilongjiang Province, Regional geology of Heilongjiang Province, Geology Press, 1993.

In addition, there are also granitoid intrusions and polyphasic vein rocks, which are composed of Indo-Chinese epoch biotite granite-biotite adamellite, Yanshan epoch monzogranite-moyite, andvein rocks such as diorite porphyrite, diorite, granodiorite and lamprophyre which intruded after Indo-Chinese epoch granites.

### 2.2. Geologic structure

There are 14 composite volcanoes in Wudalianchi, which are still active until the Quaternary period. Shown by aeromagnetic data, there are NW and NE trending faults [30], see Fig 2. Faults and intrusions of magma resulted in many uplift structure and depression structure areas, the Cretaceous deposits in the depressed areas are thick, with an average thickness of about 300 m.

### 2.3. Volcanic eruption cycle

The seven eruption cycles of the Wudalianchi volcano group are as follows, Gelaqiushan eruption cycle (2.07 million years ago), Wohushan eruption cycle (1.05 ~  1.42 million years ago), Jiaodebu eruption cycle (700,000 ~  800,000 years ago), Weishan eruption cycle (400,000 ~  500,000 years ago), Bijiashan eruption cycle (280,000 ~  340,000 years ago), Xilongshan eruption cycle (170,000 ~  190,000 years ago) and Laoheishan eruption cycle (1,719 ~ 1,721 years ago). Because the drilling holes we collected are most distributed in the strata formed by the first six eruptions, whereas few of drilling holes are collected in the seventh eruption-Laoheishan eruption, so the first six eruptions are taken as one stratification while the Laoheishan eruption is taken as another.

## 3. Data and methods

### 3.1. Input data

It is the most effective and reliable method to construct 3D geological modeling by using borehole data to interpolate geological surfaces [31–34]. The input data used in the model are investigative data of 46 wells, including artificial well data in 2019, provincial construction well data in 2020–2021, automatic groundwater well data in 2019 and field investigation well data in 2020, among which the deepest well reaches 164 m and the shallowest well is 12 m. Besides, 14 complex volcanoes are modified with data from geological points and DEM data. DEM data can describe the volcanic landform of Wudalianchi in detail, especially the volcanic cone, crater, lava platform and volcanic barrier lake, and provides data support for the establishment of three-dimensional geological structure model of Wudalianchi volcanic area.

### 3.2. Modeling program

The application of GMS in modeling is flexible, and it can be modeled in different data modes, such as borehole data, scatter data, etc., which meets the requirement of multiple modeling methods for hierarchical modeling. GMS Modules involved in this modeling are Boreholes, Map, UGrid, 2D Scatter Point, TIN and Solid. Boreholes Module constructs 3D cross sections between boreholes bases on the stratigraphic data. In the Boreholes Module, horizons modeling method is implemented by assigning a different layer code to each section of the given formation is the most basic and widely used method for 3D geological modeling based on borehole data [35]. The Map Moduleis used to create feature objects on the image in order to represent boundaries and structures, which is a foundation for the construction of conceptual model. UGrid Module is the latest module in GMS and is used with unstructured grid geometric objects. Unstructured grids are very flexible, they can include many types of cells including 2D and 3D cells and cells with any number of faces and nodes. The stratigraphic characteristics of volcanic areas are the result of comprehensive process including diagenesis, composition, weathering [36] as well as tectonism [37], which are different from those of sedimentary rock areas. While the unstructured grid of UGrid Module can more realistically reflect geological characteristics of volcanic areas such as lithological pinch-out, pinpoint and so on.

**Fig 3:**
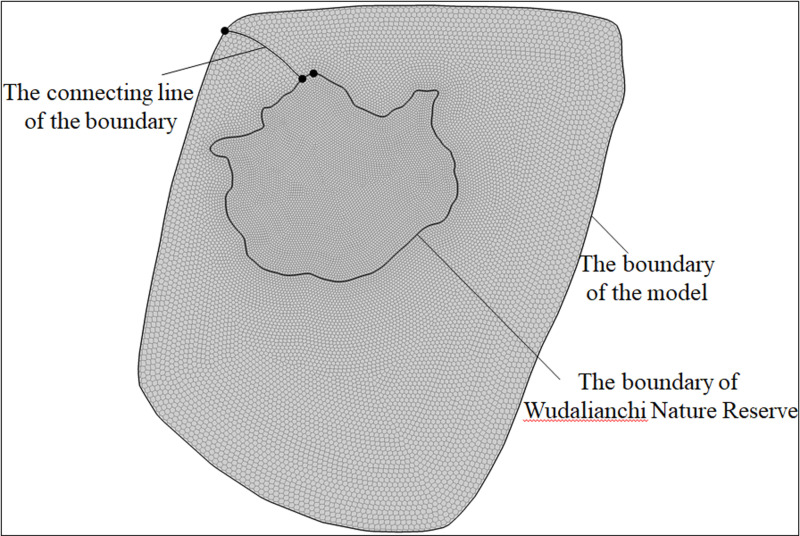
Ugrid voronoi graph of the simulation area.

**Fig 4:**
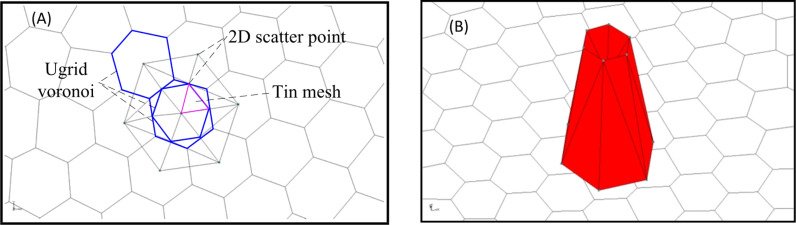
Schematic of building volcanic cone. (A) XY plane diagram of volcanic cone. (B) 3D diagram of volcanic cone.

Unlike water flow and water quality models, it is necessary to adjust parameters to fit the measured data and simulated data, so as to verify the rationality of the model. In the construction of geological models, the most important for the setting of key data (in addition to basic data) is the number of boundary divisions, which will directly affect the calculation time, and even pause the calculation. The author conducted a proper statistical analysis on the number of boundary splits and the selection of boreholes from cell centers. The number of boundary splits used to be set at 100, 200 and 300 segments. When the number of boundary splits was set at 300 segments, the calculation time was significantly increased compared with 200 segments. So the boundary of Wudalianchi Nature Reserve and the modeling boundary are both divided into 200 segments, and the connecting lines of the two boundaries are divided into 15 segments. Then, the Map Data is transformed into 2D UGrid. The Voronoi whose mesh is refined around points that are marked as refine points based on the specified segmentation section number and constructed to honor all arc geometry is used in UGrid type (Fig 3). Of course, there are many other UGrid types as well, like Regular (not refined), Quadtree/Octree and Nested Grid. Voronoi is chosen because the polygons it creates can be decomposed into multiple triangular grids based on the locations of cell centers. That is, the TINs converted from 2D Scatter points can be seamlessly connected to UGrid (Fig 4A). 2D Scatter Point Module is used to interpolate from groups of 2D Scatter data to other objects (meshes, grids, TINs). Several interpolation schemes are supported, including kriging. In this study, 2D Scatter Point Module is used to create objects (TINs) from the scatter data for establishing 14 composite volcanoes. Finally, TIN is transformed into UGrid to realize the hierarchical construction of 3D geological structure model (Fig 4B). During the transformation of 2D Scatter points into TIN, the TIN material and Horizon ID can be set through the properties dialog, which provides possibility for the construction of composite volcanoes. Meanwhile, TIN can be used to fill the composite volcanoes so as to generate solid mode, the create cross section in which can establish solid cutting model of any section.

### 3.3. Strata generalization

According to the actual stratigraphic distribution in the area, the strata are generalized into six lithologies, which are respectively designated as clay-1, sand-2, sandstone-3, mudstone -4, intrusive -5, basalt -6. The basalts are divided into two parts: the lower part and the upper part. The lower basalts are mainly composed of the lower and middle Pleistocene basalts, pumice clasts, volcanic bombs, volcanic cinder and fused breccia lumps with loose deposits, while the upper basalts are mainly composed of the Holocene basalts (the basaltic platform exposed to the surface). The intrusive rocks are Indosinian and Yanshanian granites. The mudstone is the upper Cretaceous Nenjiang Formation mudstone and silty mudstone. The sandstone is fine grained sandstone and conglomerate in the Nenjiang Formation of the Upper Cretaceous. The sand consists of middle and lower Pleistocene sand and gravel, medium and fine sand mixed with silty clay. The clay is Holocene and Upper Pleistocene silty clay, mucky silty clay and sandy silty clay.

### 3.4. Hierarchical establishment processes of the 3D geological model

Based on the borehole data, we strips the top clay layer to built the first layer composed of Cretaceous sandstone and mudstone, Quaternary sand, intrusive rock, volcanic lava from the eruption of old volcanoes, and the loose deposits among them(referred to as early basalt). The second layer is the construction of ancient composite volcanoes. The third layer is the Quaternary clay layer. The fourth layer is the latest composite Laohei volcano and Huoshao volcano, as well as the basalt platform formed by eruption (referred as late basalt). The geological model can be constructed layer by layer from the bottom to the top.

#### Construction of the first layer.

**Fig 5:**
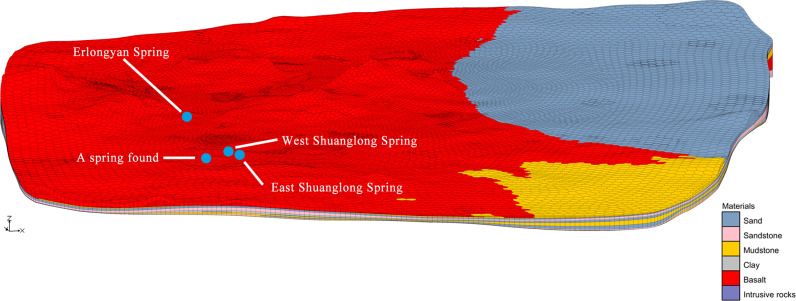
The first layer of 3D geological map (Z magnification: 20).

Except the Quaternary clay beds, other rock strata are used to create a 3D geological structure of Cretaceous sandstone and mudstone, Quaternary sand, intrusive rock and early basalt beds (Fig 5). In the calculation of horizons to UGrid, top of borehole and bottom of borehole are chosen as the top and bottom elevations of the model, respectively. Besides representing missing horizons implicitly was selected. The interpolation method used in the establishment of any horizon UGrid is inverse distance weighted, which is suitable for the areas with faults, hiatus of formations, and uneven distribution of boreholes [38]. The most commonly used form of inverse distance weighted interpolation is the constant nodal function sometimes called “Shepard’s method”. (The interpolation results of other two node functions have been verified that the thickness of the rock layer will be distorted.

Shepard’s Method is used for modeling based on 46 boreholes data. In the processes of modeling, if the boreholes are distributed around the volcano and close to the volcanic cone, the roof elevation of the basalt formation will be higher than they actually are. The main reason is that, under the influences of topography and magmatic activities, the rock strata formed by volcanic eruptions is thicker in the local range near the volcanic cones, and the farther away from the cones, the thinner the thickness becomes until they pinching out. However, when the boreholes are close to the volcanic cone, the interpolation algorithm will automatically raise the surrounding elevation under the control of the elevation of the volcanic cone, which will naturally increase the thickness of basalt layer and distort the geological structure model. If the boreholes are located far away from the volcanic cone (this study found that most of the boreholes are located far away from the volcanic cone), the height of the formation roof is lower than the height of the volcano. Therefore, on the basis of the above first layer modeling, it is necessary to use geological points data to separately construct the ancient composite volcanoes so as to correct the thickness of the basalt formation.

#### The establishment of ancient composite volcanoes.

**Fig 6:**
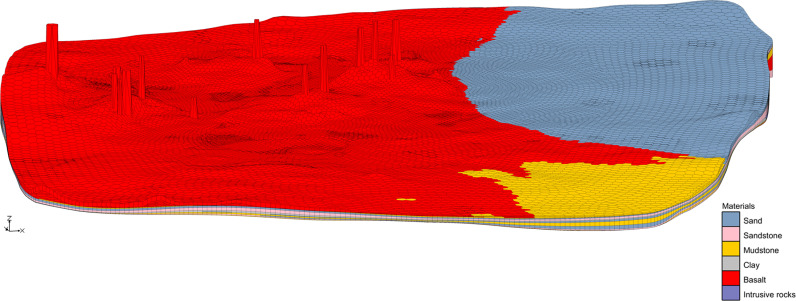
Distribution of old complex volcanoes (Z magnification: 20).

The 12 ancient composite volcanoes are summarized in Table 2. The information in Table 2 are converted into 2D Scatter point format (X, Y, Dataset) with a total of 200 scattered points based on DEM data and volcanic cone range (of course, the more the scatter points are converted, the truer the correction of volcanic cones are). The scattered points of each cone are individually imported into the model, and the scattered data is converted into TIN. The reason why we choose TIN here is that we can select the Material and Horizon ID for this layer separately through TIN Properties and then convert the TIN into the UGrid, which will achieve a full fit with the first layer. Fig 6 shows the 3D geological structure model after the construction of the ancient composite volcanoes.

**Table 2.** Overview of old complex volcanoes.

Cone		Altitude (m)	Base diameter(m)	Cone slope	Crater inside diameter(m)	Crater depth(m)
Wei volcano		518	89	20 ~ 30°	350	89
Molabu volcano		524	780		250	44
East Longmen volcano		578	900		380	101
West Longmen volcano		548	900	25 ~ 40°	300	134
Xiaogu volcano		459	650		400	31
East Jiaodebu volcano		545.3	780		350	38
West Jiaodebu volcano		482	600		240	22
South Gelaqiu volcano		602.6	1,000	30°	470	50
North Gelaqiu volcano		543	500		230	
Bijia volcano		507.8	760			63
Yaoquan volcano		357.7	550	20°	230	37
Wohu volcano	North of the cone	498.5	1,450		320	36
	South of the cone	465	600		350	17
	West of the cone	479	600		250	19
	East of the cone	493.5	1,450		240	10

*Note: According to Science Technology Commission of Heihe District and Dedu County in Heilongjiang Province, Volcanic geology and comprehensive utilization of mineral resources in Wudalianchi, 1974.

#### The establishment of Quaternary clay beds.

**Fig 7:**
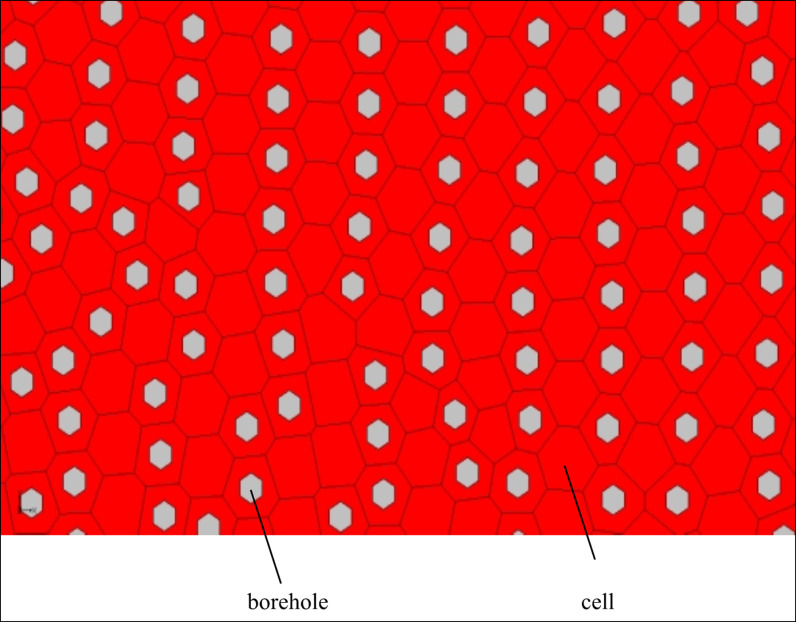
Borehole distribution diagram in a part of simulation area.

**Fig 8:**
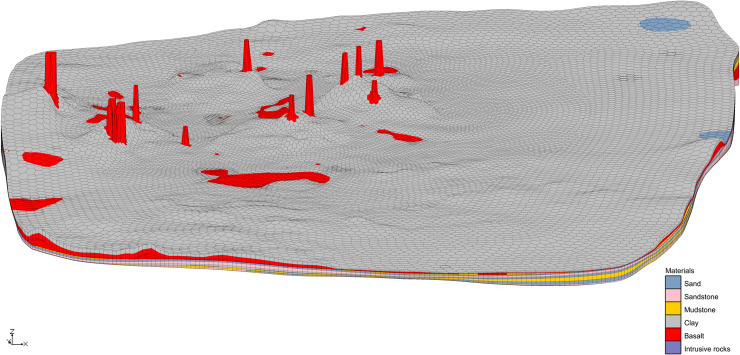
Diagram of clay layer modeling (Z magnification: 20).

The clay bed is constructed in the form of drillings, and the lower elevation of the drillings is the upper elevation of the first layer we just established. Under the premise of ensuring that the clay layer and the first layer are as close as possible, and without excessive calculation (too many drilling holes), a total of 9,824 drilling holes are selected by Cell Centers (Fig 7 shows the distribution diagram of some boreholes in the simulation area). The coordinate data of these boreholes are added in batch through ArcGIS, and then the DEM data are extracted to points, and finally the upper elevation of each borehole is derived. The Quaternary clay layer is constructed through Shepard’s Method using the upper and lower elevation data of boreholes (9,824 boreholes are selected and 46 are extant, a total of 9,870) (Fig 8).

#### The construction of late basalt platform.

**Fig 9:**
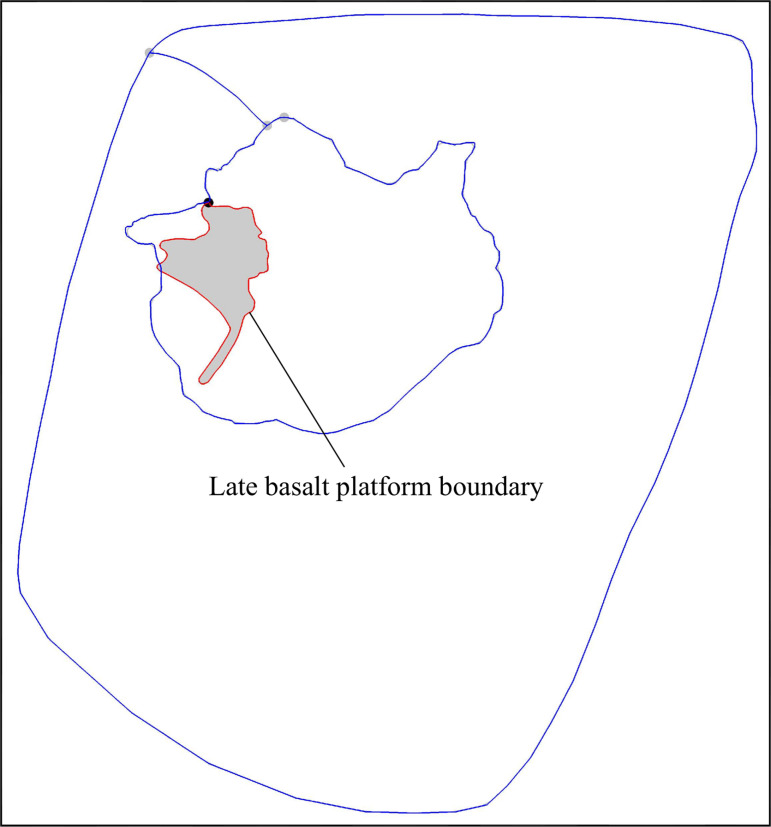
Distribution map of late basalt platform (According to Administration Committee of Wudalianchi Scenic Area, Scope and function zoning map of Wudalianchi National Nature Reserve in Heilongjiang Province with a scale of 1:145000, 2017).

**Fig 10:**
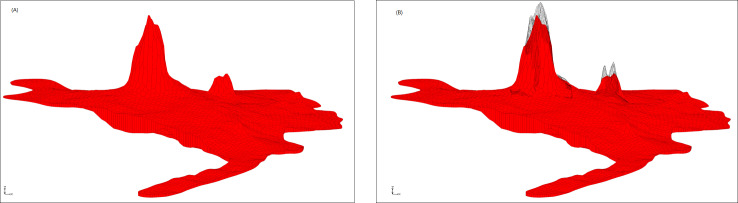
3D geological map of late basalt platform. (A) 3D geological map based on borehole data. (B) 3D geological map modified by scattered points.

The Laohei volcano basalt is exposed in Laohei volcano and Huoshao volcano, covering an area of about 68.15 km^2^, accounting for 14.37% of the volcanic stratigraphy. The boundary of late basalt platform is imported to the above model boundary, as shown in Fig 9. The establishment of basalt platform is based on the Borehole Module and the 2D Scatter Module. 541 boreholes are selected in late basalt platform (The boreholes selected as the control point must be able to control the shapes of the volcanic cone, crater and the surrounding basalt platform. Therefore, the extent of the late volcanic lava eruption is determined based on the regional topographic map, and then the control points can be determined), of which the bottom elevations are the upper elevation of the clay layer, and the top elevations could be extracted from ArcGIS based on DEM data. Through calculations from Horizons to UGrid, we can establish late basalt platforms (Fig 10A). It can be seen that morphological characteristics of Laohei volcano and Huoshao volcano established using drilling data are much truer than previously established. But limited to the amount of calculation and the number of drilling, the Laohei volcano and Huoshao volcano don't tally well with the actual situation (loss of elevation control points), thus we need to add 2D Scatters to fix the rue form of volcanoes. According to the volcano profiles and the DEM, a total of 2,972 scattered points are added. The modified volcanoes are shown in Fig 10B. We can see that the corrected Laohei volcano and Huoshao volcano are higher in elevation than that in Fig 10A at some points.

**Fig 11:**
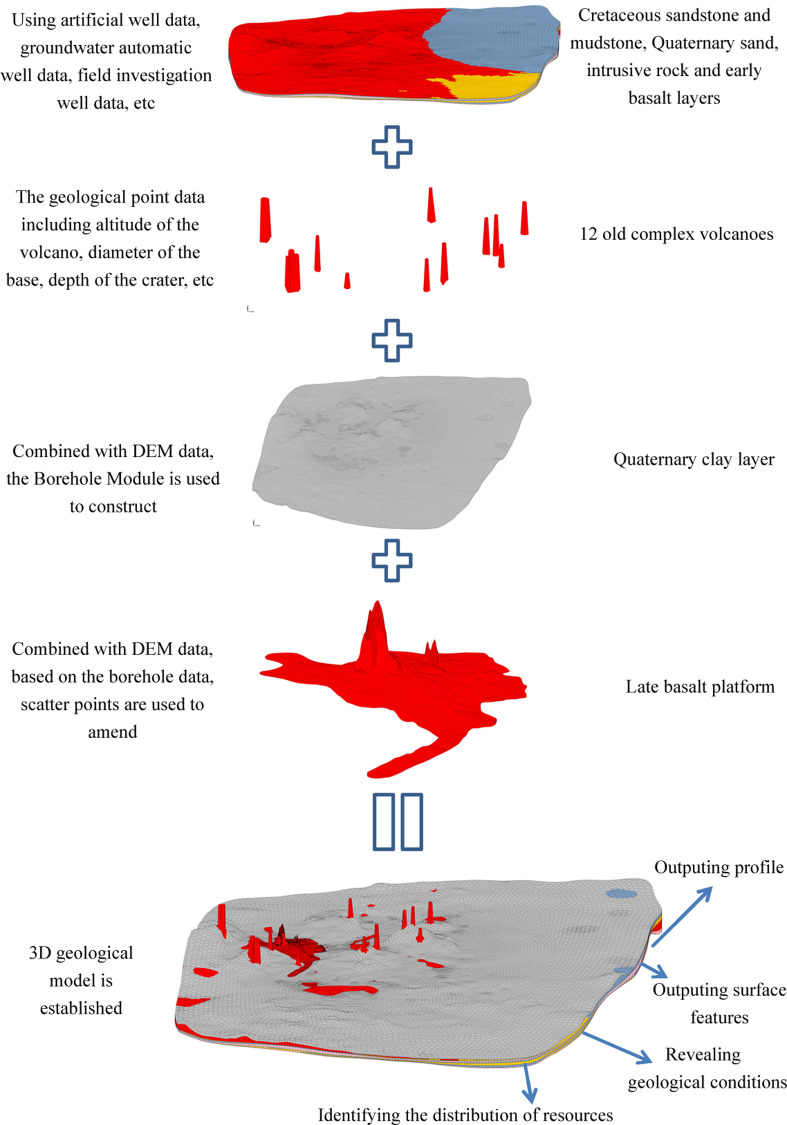
The sketch illustrating establishment process of 3D geologic structure model ofWudalianchi volcanic area.

To sum up, the establishment processes of 3D geological structure model in Wudalianchi volcanic area are shown in Fig 11.

## 4. Model validation

**Fig 12:**
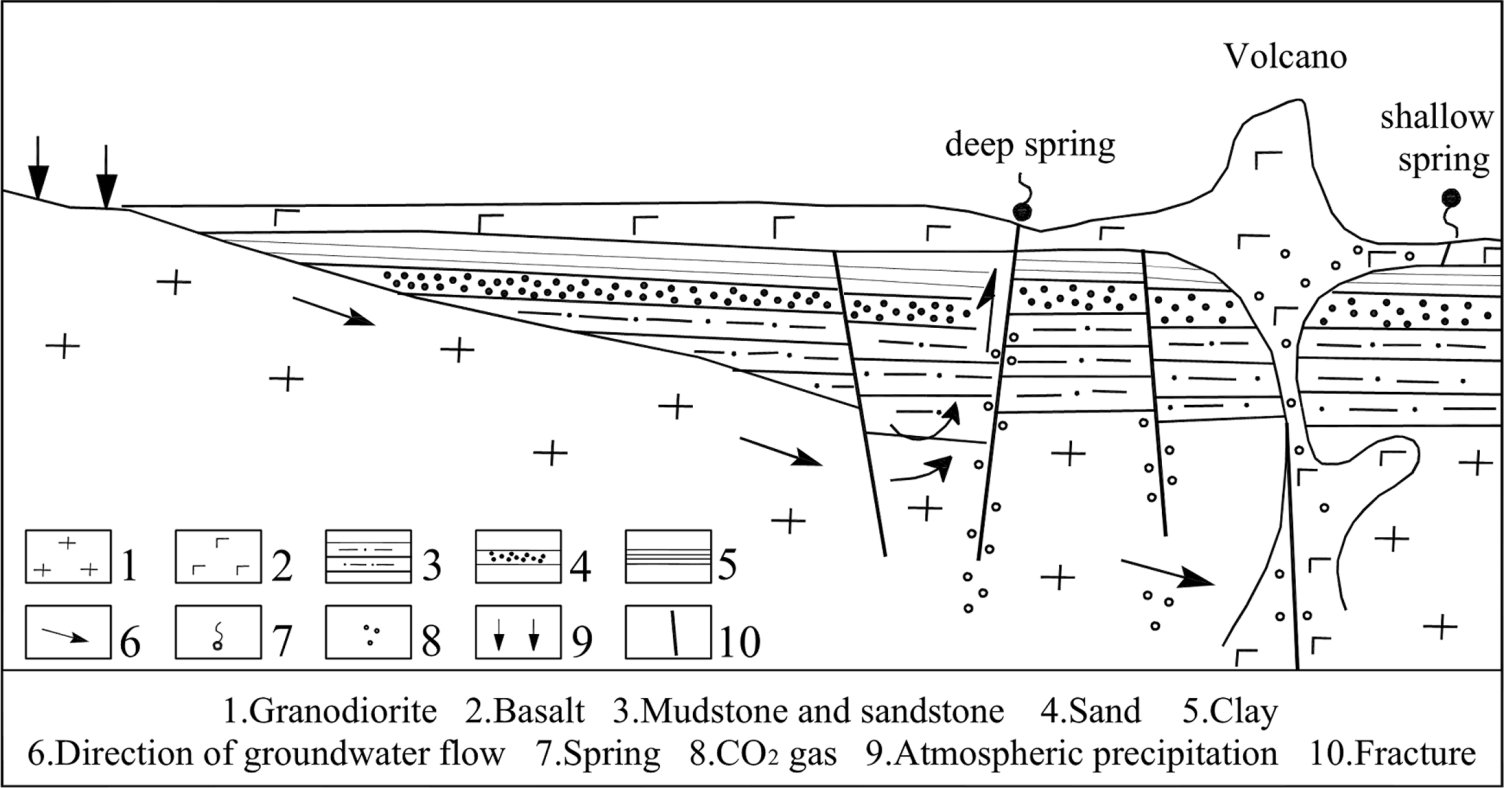
The formation mechanism of shallow Spring and deep Spring.

The validation of 3D geological structure model is always a difficult problem, especially in areas lack of profiles. In this paper, the formation mechanism of deep springs, shallow springs (Fig 12) and the Baolong spring are used to verify the 3D geological structure model of the volcanic area.

The outcrops of springs are mainly controlled by the geomorphic conditions. Most of the shallow springs are distributed on the edge of lava platform with relatively low elevations, belonging to phreatic water in the Quaternary basalt fissure, such as the Erlongyan Spring, the East and West Shuanglong Spring, and a spring found in Qingquan Village during field investigation (Fig 5), which are distributed in the southern edge of the basalt platform. This is mainly controlled by the flow direction of groundwater in the basalt platform from northeast, northwest to south -central.

**Fig 13:**
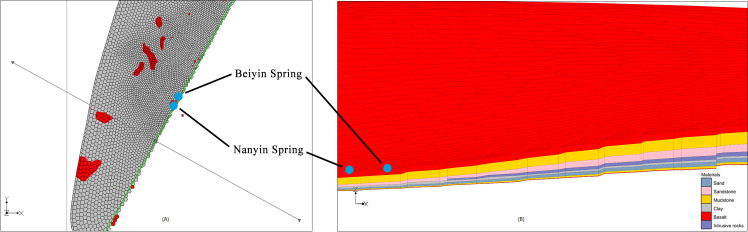
Section diagram of Nanyin Spring and Beiyin Spring. (A) Position diagram of section. (B) Stratigraphic distribution map of section.

Besides the above shallow springs, there are also deep springs from confined water affected by faults in this area. The outcroppings of the Nanyin Spring and Beiyin Spring are mainly controlled by the deep north-south fault. Phreatic water in basalt fissure and deep overflowed mineral water at the same time overflow the surface in the north-south fracture, forming two secondary mineral springs-Nanyin Spring and Beiyin Spring. According to the flow direction of groundwater (from north-east to south-west), the reliability of the model is verified by selecting a section where the Southyin and Northyin springs are located (see Fig 13A, the green line is the profile line). The stratigraphic distribution of the section are shown in Fig 13B, the intrusive rocks formed through the activities of the ancient composite volcanoes-East Jiaodebu and West Jiaodebu do not extend to the location of two springs, whereas pinch-out at the northeast of the two springs. This agrees with the actual distribution of the intrusive rocks (Han Jianchao et al., 2012 [39], Schematic diagram of groundwater circulation in mine water area of Yaoquan Mountain). It can also be seen from the profile that the two secondary mineral springs are mainly composed of cretaceous sandstone fissure-pore water and Quaternary basalt fissure water. Moreover, the Cretaceous sandstones and mudstones are thick, which is consistent with the geological background belonging to a depression area near Yaoquan Volcano. Therefore, the reliability of the model can be verified by this section.

**Fig 14:**
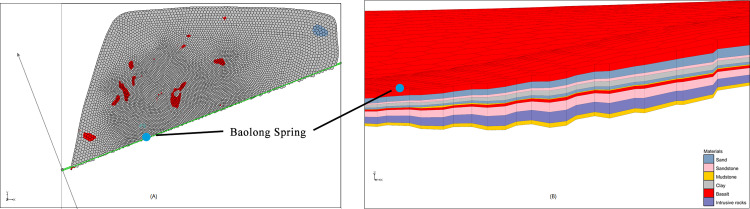
Section diagram of Baolong Spring. (A) Position diagram of section. (B) Stratigraphic distribution map of section.

Mineral springs in Wudalianchi are well known not only for bicarbonate- carbonate mineral water, but also for radon mineral water with medical value (it can treat a variety of chronic diseases). Radon gas comes from the decay of Ra belonging to the offspring of U and Th enriched in deep granite. Previous explorations have confirmed that radon mineral water exists in the granite fissured confined aquifers at Yaoquan Volcano, with the content of radon up to 1,300 Bq/L. According to the survey of springs and wells, the highest radon contents of Nanyin Spring and Beiyin Spring are up to 55 Bq/L and 37 Bq/L, while Baolong Spring has a higher radon content and can meet the standard of radon mineral water [40]. It can be seen from the profile of Baolong Spring (Fig 14) that the thickness of the deep granite is greater than the selected section of the Nanyin and Beiyin Spring, and it continues to the emerging position of Baolong Spring along with the direction of underground flow (from northeast to southwest), that’s why the radon content in Baolong Spring is higher. The Cretaceous sedimentary strata revealed by Baolong Sping section is thinner than those revealed by Beiyin Spring and Nanyin Spring section, which is consistent with the actual stratigraphic distribution.

## 5. Conclusions and prospects

The following conclusions are drawn from the establishment processes of the 3D geological structure model:

(1) Influenced by the multi-stages of volcanoes eruption, the thickness of rock strata around multiphase volcanoes is less difference than that formed by a single eruption, especially the basalt layer, which provides the possibility for the application of inverse distance weighted interpolation and layered modeling.
(2) The unstructured grid of UGrid can better reflect the real stratigraphy in the volcanic region. TIN Module should be applied in the establishment of volcanic cones, because it can support the selection of triangle face materials composed of scattered points, which provides necessary conditions for the establishment of volcanic cones.
(3) Modeling with drilling data will not generate additional boundaries, whereas with scatter points, an additional closed boundary will be automatically generated due to the connection lines of scatter points. However, in order to accurately capture the volcanic control points, improve the computational efficiency, and establish and modify the volcanic morphology, it is necessary to select scatter points to establish the 3D model. Therefore, in the processes of modeling, we should choose the appropriate modeling method according to the actual situation.
(4) The formation of spring is mainly affected by the comprehensive effects of topography and geological conditions. Therefore, along the flow direction of the spring, sections can be selected to verify the distribution of rock strata and thus demonstrate the reliability of the structural model.

On the basis of this study, the following prospects are put forward for the construction of 3D geological structure model in volcanic areas:

(1) The hierarchical construction provides the possibility for reproducing the stratigraphic structure of volcanic area where the data is scarce and the stratigraphic distribution is complex, as well provides an effective method and a demonstration role for the construction of 3D geological structure model in volcanic zone.
(2) The establishment of 3D geological structure model of Wudalianchi volcanic area can lay a foundation for the prediction and evaluation of regional groundwater, geothermy, mineral water and mineral mud resources, and provide theoretical basis and technical supports for sustainable utilization of regional resources.
(3) There are abundant surface water and groundwater resources in the area, and the conversion between them is frequent. The establishment of geological structure model can provide scientific basis for the research on the conversion of surface-groundwater water, the mechanism of basin production and confluence, the construction of water source area and the security of water environment and ecosystem.
(4) Given the limited drilling data and geological points, the geological structure model can’t represent the regional stratigraphic distribution exactly, therefore, we should increase the density and the depth of drills, gather more geological points to establish a geological structure model approximating the real state, and validate the model with known geological information such as geological points, profiles, and springs.
